# Full elastic strain and stress tensor measurements from individual dislocation cells in copper through-Si vias

**DOI:** 10.1107/S2052252515015031

**Published:** 2015-09-30

**Authors:** Lyle E. Levine, Chukwudi Okoro, Ruqing Xu

**Affiliations:** a Materials Science and Engineering Division, National Institute of Standards and Technology, 100 Bureau Drive, STOP 8553, Gaithersburg, Maryland 20899-8553, USA; b Semiconductor and Dimensional Metrology Division, National Institute of Standards and Technology, Gaithersburg, Maryland 20899-8120, USA; c Advanced Photon Source, Argonne National Laboratory, Argonne, Illinois 60439-4800, USA

**Keywords:** full elastic strain, stress tensor measurement, copper through-Si vias, microelectronics

## Abstract

Nondestructive measurements of the full elastic strain and stress tensors from individual dislocation cells distributed along the full extent of a 50 µm-long polycrystalline copper via in Si is reported. Determining all of the components of these tensors from sub-micrometre regions within deformed metals presents considerable challenges. The primary issues are ensuring that different diffraction peaks originate from the same sample volume and that accurate determination is made of the peak positions from plastically deformed samples. For these measurements, three widely separated reflections were examined from selected, individual grains along the via. The lattice spacings and peak positions were measured for multiple dislocation cell interiors within each grain and the cell-interior peaks were sorted out using the measured included angles. A comprehensive uncertainty analysis using a Monte Carlo uncertainty algorithm provided uncertainties for the elastic strain tensor and stress tensor components.

## Abstract 2

A ground breaking new capability for measuring complete strain and stress tensors nondestructively from deeply buried, sub-micrometre sample volumes within microstructurally complex and multicomponent specimens is presented. The method is demonstrated on technologically important copper through-Si vias that are used in advanced three-dimensional microelectronics.

## 1. Introduction   

Recent advances in synchrotron X-ray techniques (Larson *et al.*, 2002 ▸; Yang *et al.*, 2003 ▸; Ice *et al.*, 2005 ▸, 2011 ▸; Jakobsen *et al.*, 2006 ▸, 2007 ▸; Levine *et al.*, 2006 ▸, 2011 ▸) have provided an unprecedented capability for probing phases, microstructure, and elastic strains with micrometre, and even submicrometre, spatial resolution within complex real-world materials (Larson & Levine, 2013 ▸). For example, recent experiments on 34ID-E of the Advanced Photon Source (APS), Argonne National Laboratory, used energy-scanned Laue diffraction with depth-resolved, submicrometre-focused X-ray beams to measure the axial 006 lattice parameter of isolated large dislocation cell interiors in heavily (30%) deformed copper (Levine *et al.*, 2006 ▸). In later work, improved instrumentation and techniques allowed Levine *et al.* (2011 ▸, 2012 ▸) to measure diffraction line profiles from numerous *individual* dislocation cell walls and cell interiors within a heavily deformed Cu single-crystal, and even from an ultrafine-grained commercial Al alloy that was severely deformed using equal-channel angular pressing (Lee *et al.*, 2013 ▸; Phan *et al.*, 2014 ▸).

These studies provided groundbreaking results, but they were limited to just a single reflection. Thus, only a single component of the elastic strain could be measured. Ultimately, a primary, long-range goal of that microbeam diffraction facility is to enable full strain and stress tensors to be extracted from buried submicrometre-scale sample volumes within complex devices and microstructures. We report the measurement of full elastic strain tensors from individual dislocation cell interiors distributed along the 50 µm length of a copper through-Si via (Cu-TSV). These strain tensors are then converted to full stress tensors using the known elastic constants for Cu. Measurement uncertainties were propagated through the experimental design using a Monte Carlo uncertainty algorithm to provide uncertainties for each strain and stress component.

The determination of stresses within Cu-TSVs is important for microelectronic applications, and preliminary results have already been reported separately (Okoro *et al.*, 2014*a* ▸,*b* ▸). Here, the primary emphasis will be a detailed description of the measurements and analysis required to determine the strain and stress tensors, and a thorough discussion of the uncertainty analysis.

## 2. Experimental   

**Figure 1:**
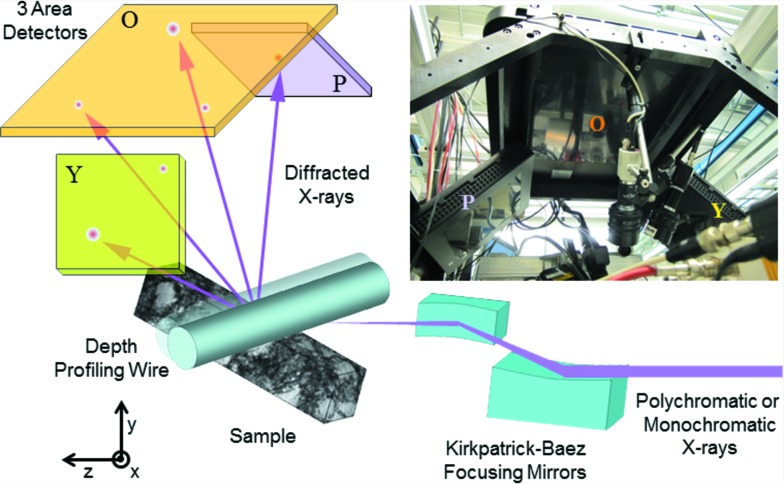
Diagram of the microbeam diffraction instrument showing all major components discussed in the text. The upper right corner is a photograph of the area detectors as seen from below and the laboratory-frame coordinate system is shown in the lower left corner. The *x* axis extends out from the drawing.

Fig. 1 ▸ is a diagram of the microbeam diffraction instrument on APS sector 34ID. The X-ray beam from the insertion device is followed by a translating monochromator and vertical and horizontal slits, producing either a polychromatic or monochromatic X-ray beam that is shown entering from the right of the diagram. The beam is then focused using an orthogonal pair of elliptically figured Kirkpatrick–Baez focusing mirrors. In these measurements, the focused beam size was approximately 1.0 µm vertical × 0.6 µm horizontal. Diffracted X-rays from the sample are incident upon three amorphous Si area detectors with 200 µm square pixels. The large central detector (designated ‘O’ for orange), has 2048 × 2048 pixels and is roughly centered over the sample; the two smaller side detectors (purple and yellow) have 1024 × 1024 pixels and are tilted to provide the maximum angular acceptance for the diffracted X-rays. A 50 µm-diameter Pt wire can be inserted to provide depth profiling. This wire is incrementally translated parallel to the sample surface as images are acquired from the area detectors. By subtracting successive images, the X-rays blocked by the profiler during each step are identified and the corresponding sample depths are obtained by triangulation. A more thorough description of this procedure is given by Levine *et al.* (2011 ▸).

Polychromatic measurements of a single reflection only provide information on the direction of the diffracted X-rays with respect to the incident beam. Energy-scanned measurements are much slower, but they also allow the lattice parameter to be determined. The full strain tensor can be obtained by conducting energy-scanned measurements using at least three noncollinear reflections, or by conducting at least one energy scan along with polychromatic measurements on at least four independent reflections. In this study, energy scans were conducted on three independent reflections that cover the widest possible angular range to minimize uncertainties.

In principle, this method for determining the full strain tensor is very simple, but several practical issues have prevented this method from being employed successfully so far. Firstly, the measured reflections must all originate from the same sample volume for the strain tensor to be meaningful. Ideally, the depth-resolving wire should allow such volumes to be determined. However, as widely spaced reflections intersect the wire at positions up to a few millimetres apart, even small uncertainties in the wire shape, position and motion can make it impossible to determine the true depths with adequate accuracy. Secondly, when a sample is plastically deformed, the diffraction spots become smeared out and the angles between the reflections can no longer be determined with sufficient accuracy to provide meaningful tensor components (Larson & Levine, 2013 ▸). Finally, once these problems are solved, it is also critical to perform a rigorous uncertainty analysis to determine how the experimental uncertainties affect each of the extracted tensor components.

**Figure 2:**
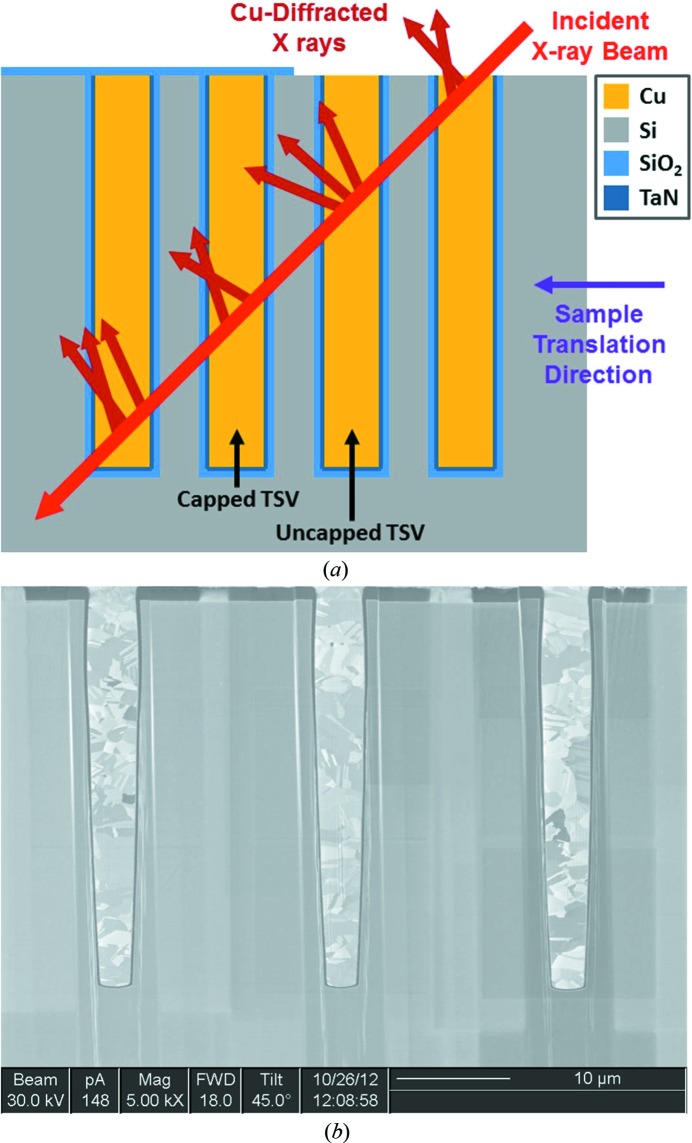
(*a*) Geometry of Cu-TSVs along with the incident X-ray microbeam and the X-rays diffracted from the Cu. (*b*) Microstructure of typical Cu-TSVs acquired using scanning electron microscopy of cross sections cut using focused ion beams.

In this study, the origin for each reflection was primarily determined by a combination of the sample geometry and microstructure, rather than relying solely upon the depth resolving wire. Fig. 2 ▸(*a*) shows our sample geometry. For this study, blind (fully buried) Cu-TSV arrays with pitch, diameter and depth dimensions of 12 µm, 5.5 µm and 50 µm, respectively, were built into a full thickness 300 mm silicon wafer. The thickness of the isolation liner (SiO_2_) and the barrier layer (TaN) were 0.5 µm and 0.025 µm, respectively. Subsequently, the wafer was diced into separate dies; an individual die was used for this study. One hundred and seventy nanometres of plasma-enhanced chemical vapor deposited SiO_2_ was deposited on half of the die, to simulate the presence of a back-end-of-line layer, so as to study its effect on the Cu-TSV stress state. After the deposition of SiO_2_, the sample was annealed at 420°C for 30 min in a nitrogen ambient environment. The Cu-TSV described in this study was uncapped, meaning that it did not have any SiO_2_ overlayer.

The incident micro-beam enters the sample at a 45° angle on a path that intersects multiple Cu-TSVs as shown in Fig. 2 ▸(*a*). As the photon energies typically range from around 10 keV to 24 keV, the beam can easily penetrate tens of micrometres into the specimen and the diffracted intensity on the area detectors originates from several Cu-TSVs and the single-crystal Si matrix. With the beam centered on the top of the selected Cu-TSV, a wire scan was conducted using polychromatic X-rays and all three area detectors. The resulting images were reconstructed into depth-resolved white beam Laue patterns as described previously (Levine *et al.*, 2011 ▸, 2012 ▸), thereby identifying the diffraction peaks that originate from the target Cu-TSV.

Fig. 2 ▸(*b*) is a scanning electron microscopy image of a focused ion beam cross section of a randomly selected die. The Cu-TSVs are clearly polycrystalline, with grain dimensions ranging from much less than 1 µm up to at least 6 µm. Thus, several different grains typically contribute to the measured diffraction pattern.

After indexing the white beam Laue patterns from the targeted Cu-TSV, a single grain was selected with clear spots on all three detectors. A single bright reflection on each detector was then chosen so as to cover the largest possible angular range. An energy scan was conducted on each selected reflection over an energy range (up to about 400 eV) large enough to include the full range of crystallographic orientations present in the grain. The energy step size was set to 3 eV to match the microbeam convergence.

Following the energy scans, the sample was translated parallel to the sample surface so the incident X-ray beam would intersect the targeted Cu-TSV 5 µm deeper along the via axis, and the full set of polychromatic and monochromatic measurements was repeated. This procedure was repeated until diffraction data were obtained from the entire 50 µm length of the Cu-TSV.

## 3. Analysis methods   

**Figure 3:**
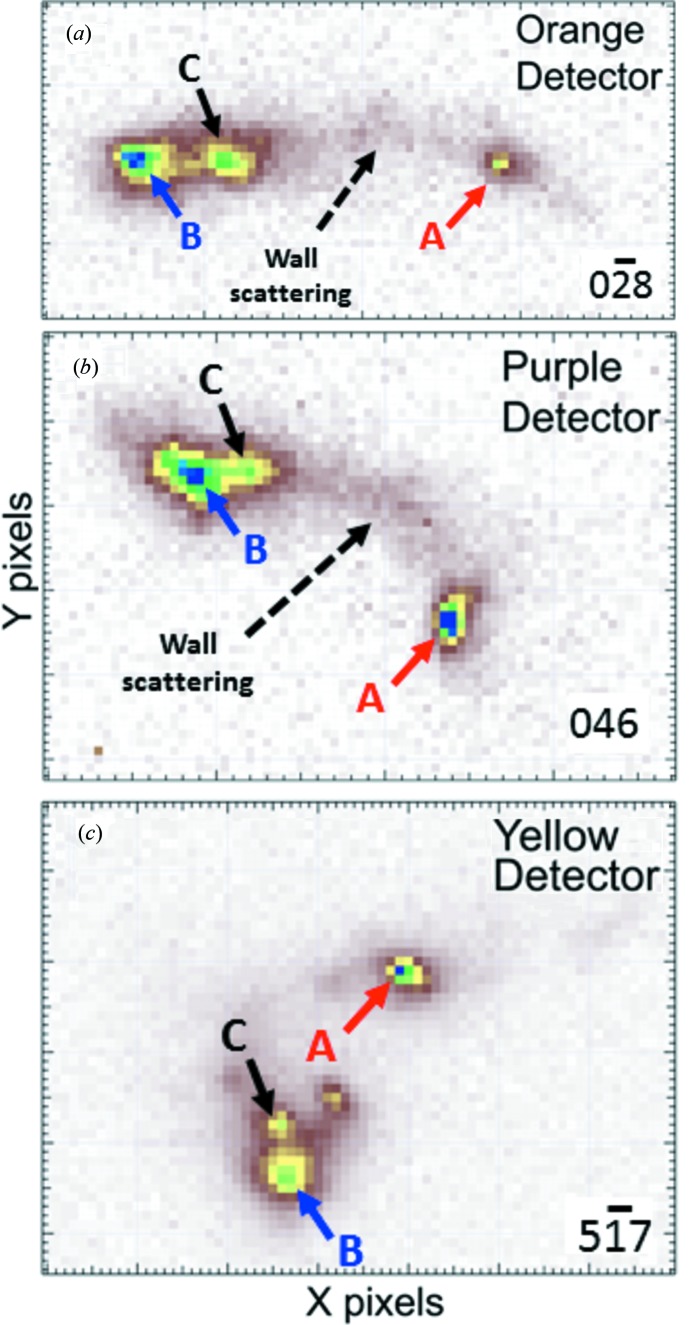
Energy-integrated distribution of diffracted X-ray intensity on the area detectors from a single grain in the Cu-TSV: (*a*) 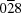 on orange, (*b*) 046 on purple and (*c*) 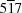 on yellow.

Fig. 3 ▸ shows the energy-integrated diffracted intensity on all three area detectors from a single grain located approximately 7.5 µm deep in the Cu-TSV. Each detector exhibits sharp diffraction spots from low dislocation density volumes and diffuse scattering associated with high dislocation density. The separation of the peaks indicates that the low dislocation regions have slightly different crystallographic orientations, suggesting that the high dislocation density regions form walls that separate low dislocation density cell interiors. To set the angular scale, the subtended angle between spots A and B on the orange detector (Fig. 3 ▸ *a*) is about 0.45°. This pattern of grain breakup into distinct dislocation cells was observed in nearly all of the grains we examined, independent of their position along the via. We also note that as we are illuminating the microstructure with a microbeam, the relative intensity of the diffraction spots in Fig. 3 ▸ reflects the relative *intersected volumes*, and not the actual dislocation cell volumes.

The pattern of spot separations and intensities on the three detectors in Fig. 3 ▸ suggests that the peaks marked with A belong to a single dislocation cell interior. Comparing the expected and measured angles between these spots on the three detectors gives the results shown in Table 1 ▸. Note that all uncertainties in this paper are one standard deviation. The largest deviation from an unstrained crystal is only about 0.02°, which is consistent with all three peaks originating from a single cell interior. All other peak combinations including an A peak give dramatically larger deviations. Similar evaluations of the sets of peaks labeled B and C confirm that they also originate from separate cell interiors. In nearly all of the measurements from this via, it was found that the subtended angles between reflections were sufficient to unambiguously determine which peaks came from the same diffracting sample volume.

### 3.1. Strained unit cell and the infinitesimal strain tensor   

As mentioned above, energy scans of three widely separated, independent reflections are sufficient to extract a complete strain tensor, and the subtended angles between multiple sub-peaks can be used to identify a set of reflections that originate from the same sample volume. We proceed by first determining the location of each selected peak on its detector. The peak center is determined using multiple methods including visual inspection, fitting the peak with a Gaussian surface, and finding the center-of-mass using several detector areas around the apparent peak center. In most cases, all of the resulting peak positions are in good agreement and the small uncertainties are estimated from the spread. However, the peaks are sometimes asymmetric, making automatic peak fitting unreliable. Until adequate routines are developed and validated, each peak fit must be individually inspected and the uncertainties expanded if necessary.

As described previously (Levine *et al.*, 2011 ▸), an energy scan gives us the X-ray intensity incident upon each pixel on each detector as a function of photon energy (or wavelength). Since the position of each pixel is known through the instrument calibration, the energy-dependent intensities can be converted into a diffraction line profile for each pixel. We then sum the line profiles from all of the pixels associated with a given peak on the detector, producing a composite line profile that originates from a well defined sample volume. This composite line profile is then fitted with a Gaussian, Lorentzian or pseudo-Voigt function (as called for) to find the peak center.

**Figure 4:**
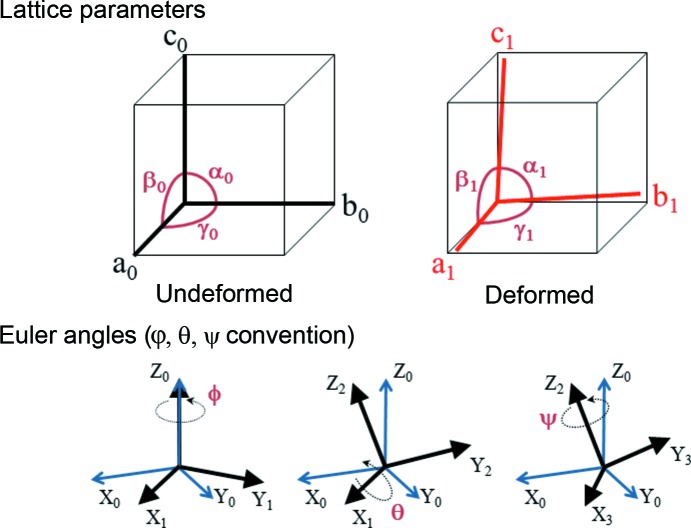
Parameter definitions describing the undeformed and deformed unit cell, and the Euler angle convention used for relating the unit-cell orientation to the laboratory-frame coordinate system.

Once the locations in reciprocal space of all three diffraction peaks are determined, the cubic lattice vectors in the laboratory coordinate system are found using a Gauss–Jordan elimination algorithm. From these vectors, the distortion of the unit cell can be directly calculated. As shown in Fig. 4 ▸, the geometry of the cubic unit cell is described using the traditional parameters *a*, *b*, *c*, α, β and γ, where a subscript of 0 or 1 denotes the undeformed and deformed unit cell, respectively.

Given the geometry of the strained and unstrained cubic unit cell, the infinitesimal (Lagrangian) strain tensor components,
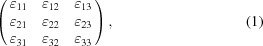
can be calculated (Schlenker *et al.*, 1978 ▸) using
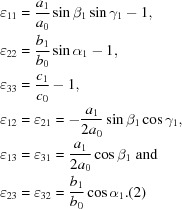



### 3.2. Euler angles   

The crystallographic orientation of the measured sample volume is important for relating the individual strain tensor components to the sample geometry and to any anisotropic external stresses or other processing conditions that may have been applied. The crystallographic orientation is described with respect to the laboratory-frame coordinate system that is shown in the lower left corner of Fig. 1 ▸. The *Z* axis points downstream along the incident X-ray beam, the *Y* axis is vertical, and the *X* axis points out from the diagram.

The orientation of the unit cell is described using three Euler angles that define a series of rotations that transform the laboratory-frame coordinate system into the crystal-frame coordinate system. All Euler angles follow the φθψ convention (sometimes referred to as the *x*-convention for the choice of the second rotation axis) described by Goldstein (1980 ▸) and illustrated in Fig. 4 ▸. The unit cell is first aligned with the [100], [010] and [001] lattice directions along the *X*, *Y* and *Z* axes of the laboratory-frame coordinate system, respectively. The unit cell is then rotated counterclockwise by φ about *Z*, giving the rotated coordinate system *X*_1_, *Y*_1_, *Z*. The next rotation is counterclockwise by θ about *X*_1_, giving *X*_1_, *Y*_2_, *Z*_2_. Finally, the unit cell is rotated counterclockwise about the *Z*_2_ axis by ψ to *X*_3_, *Y*_3_, *Z*_2_, which is the measured orientation of the deformed unit cell. The rotation matrix describing this complete transformation is 
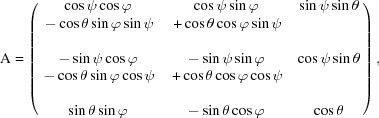
with the inverse
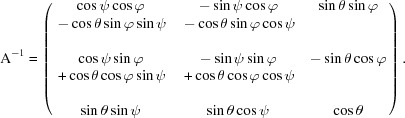
If the measured unit cell had axes that were perfectly orthogonal, the above transformation would be unambiguous. However, shear components distort these angles and an additional convention is required. Here, the reported Euler angles rotate the laboratory-frame *X* axis into the [100] direction of the measured unit cell, and the laboratory-frame *Z* axis to a direction that is orthogonal to the sample [100] and [010] directions.

The above rotation matrices can be used to easily determine the orientations of crystallographic directions in the laboratory coordinate system, and to identify what crystallographic axes are aligned along directions relevant to the sample geometry. For example, the crystallographic direction **u** points along the **u**′ = **A** **u** direction in the laboratory coordinate system. Similarly, if the vector **v**′ in the laboratory coordinate system is an important direction relevant to the sample (for example, **v**′ = [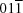] is generally perpendicular to the sample surface), the crystallographic orientation for this direction is just **v** = **A** ^−1^ **v**′.

## 4. Uncertainties   

The primary sources of uncertainty in the calculated strain tensor components include the instrument calibration, measurement uncertainties in the diffraction spot positions on the detectors, the centroid positions of the diffraction line profiles, and uncertainties in the lattice parameter of the unstrained sample. When the strain tensor is converted to a stress tensor, the uncertainties in the elastic constants must also be considered.

Instrument calibration consists of three parts: calibration of the monochromator energy, calibrations of the positions and orientations of the area detectors, and calibration of the wire position and orientation. The energy of the monochromatic beam, *E*, was calibrated using Si (*n* *n* *n*) reflections (*n* = 4–11) in a backscattering geometry, to within the energy resolution of the Si (111) monochromator, namely Δ*E*/*E* < 1 × 10^−4^. To calibrate the geometry of the detector, white-beam Laue diffraction peaks, from a 4 µm-thick strain-free single-crystal silicon calibration specimen, were collected by all three area detectors. About 50 peaks were collected and indexed on the central detector (orange), and about 10 to 20 peaks on the two side detectors (yellow and purple). The positions and orientations of the three detectors were then determined by matching the positions of the predicted and the measured peaks on the detectors through an optimization algorithm. The final root-mean-square angular uncertainties of the peak positions were ≃0.005°, which is smaller than the angle subtended by an individual pixel (≃0.02°). The orientation of the depth-profiling wire was calibrated to be perpendicular to the incident beam and parallel to the horizontal plane; this was carried out by fine-scanning the wire edges against the incident beam as well as the diffracted peaks from the aforementioned silicon specimen over a necessary range along the length of the wire. The resulting overall wire orientation had an angular deviation of less than ≃2 mrad. The origin of the wire center, most importantly its vertical position relative to the X-ray beam, was also calibrated during this process to within 1 µm.

In all cases, the calibration uncertainties are small compared with the uncertainties in the deformed copper diffraction data and they will not be included in the following analysis. It is important to note that measurements of low-defect materials such as single-crystal Si will have smaller uncertainties, and the calibration uncertainties may need to be included in the analysis.

The uncertainties in the measured peak positions and diffraction peak centers, discussed in the previous section, must be propagated through the analysis to provide uncertainties for the geometry of the strained unit cell, the Euler angles describing the crystallographic orientation, and the components of the strain and stress tensors. The strain tensor uncertainties also depend upon the uncertainty in the unstrained lattice parameter, and the stress tensor must additionally include the uncertainties in the elastic constants. As energy scans of three independent reflections comprise a minimal data set for determining the geometry of the strained unit cell and the crystallographic orientation, each such measurement produces a unique solution. Uncertainties are propagated using a Monte Carlo algorithm. Each measured value (*i.e.* the *x* and *y* positions of diffraction spots on the detectors and the centroids of the line profiles) has an associated uncertainty distribution with a standard deviation that is estimated as described above. These distributions are assumed to be approximately Gaussian in form and a set of 40 000 Gaussian-distributed variants is generated for each measured value, producing 40 000 variants of a complete measurement of three reflections. Each of these variants produces a unique solution for the strained unit cell, and the distribution of output values, such as the angle α, is analyzed to obtain a standard deviation uncertainty. Similarly, once an undeformed lattice parameter and a set of elastic constants are obtained, the Monte Carlo uncertainty propagation is carried forward for each component of the strain and stress tensors. Here, the unstrained lattice parameter is assumed to be 0.361496 (5) nm (Wyckoff, 1963 ▸) which is the lattice parameter for pure copper at room temperature. The elastic constants for copper at room temperature are c_11_ = (169.1 ± 0.2) GPa, *c*_12_ = (122.2 ± 0.3) GPa, and *c*_44_ = (75.41 ± 0.05) GPa (Ledbetter & Naimon, 1974 ▸).

As mentioned above, it is important to consider the purity of the sample because composition changes can affect the unstrained lattice parameter used for obtaining the strain and stress tensors. The elastic constants of metals are not typically as sensitive to small composition variations. As an example, in earlier work on Al alloys (Lee *et al.*, 2013 ▸), the authors used the high-resolution powder diffractometer on the APS 11-BM beamline to directly measure the ambient temperature lattice parameter of their Al 1055 alloy samples. They reported a lattice parameter of 4.05000 (10) Å as compared with a value of 4.04950 (15) Å for pure Al. This difference would produce an artificial strain of about 1.2 × 10^−4^, which is only slightly larger than the strain resolution of the microbeam diffraction instrument. In the current work, the Cu in the TSVs was deposited using an electro-plating technique, with bath additives that aid the complete filling of the trenches. The exact chemical composition of the electroplating bath is proprietary information of the manufacturer so the purity of the Cu-TSVs is not known. If a small level of impurities is incorporated as a solid solution, it would change the unstrained lattice constant (proportional to the impurity level), effectively introducing a small offset in the hydrostatic stress. Experiments on TSVs that have been allowed to relax for nine months exhibit stresses that are below 50 MPa, suggesting that any offset stress is small.

## 5. Results   

Table 2 ▸ lists all of the calculated parameters and their one-sigma uncertainties from a single dislocation cell interior located approximately 17.5 µm deep within the measured Cu-TSV. These parameters include the unit-cell dimensions and angles, the Euler angles describing the crystallographic orientation, the full strain tensor, and the full stress tensor. Note that the uncertainties vary considerably for the unit-cell parameters, and thus for the extracted strain and stress tensor components. This variation is primarily due to the angular range of the measured reflections and their orientation with respect to the unit cell. As an example, lattice vectors aligned perpendicular to the sample surface can be measured much more precisely than those with a parallel orientation.

**Figure 5:**
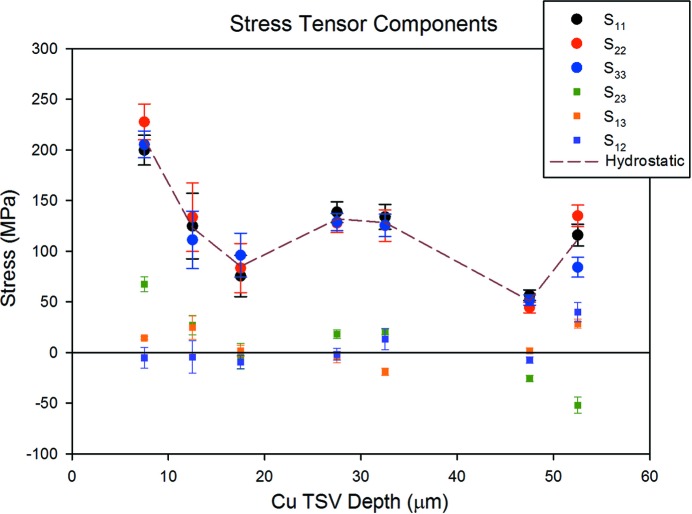
All six stress tensor components plotted as a function of depth along the Cu-TSV axis. The uncertainties are one standard deviation.

Fig. 5 ▸ shows all six stress tensor components plotted as a function of depth along the axis of the Cu-TSV. For all depths, the off-diagonal components are much smaller than the diagonal components, and the diagonal terms are approximately equal at each depth, consistent with a primarily hydrostatic stress state. The uncertainties for the diagonal components are also consistent with a hydrostatic stress state, although the overlaps suggest a slight overestimation of the uncertainties. It is also worth noting that calculating unit-cell parameters and stress tensors from individual dislocation cells within a single grain gives consistent results. For example, the lattice parameters measured in all of the cells evident in Fig. 3 ▸ vary by, at most, a strain of just 2.0 × 10^−4^ and the resulting stress state is, again, mostly hydrostatic.

**Figure 6:**
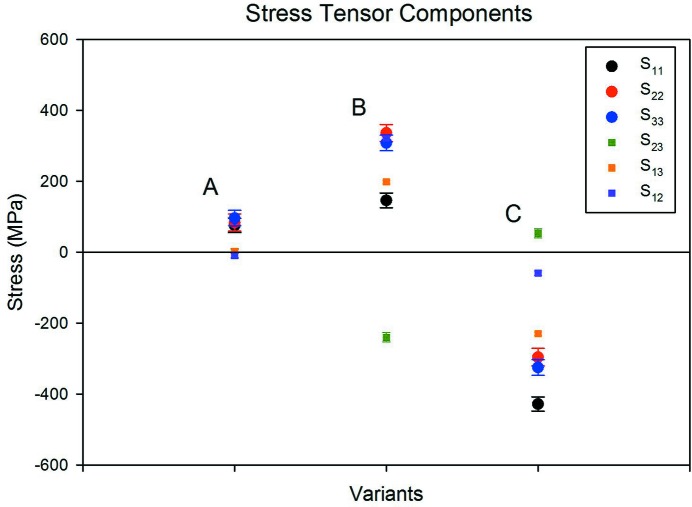
All six stress tensor components from the same sample volume plotted for three error conditions: A as measured, B first artificial peak shift, and C second artificial peak shift.

The pattern of the stress components shown in Fig. 5 ▸ provides strong supporting evidence that the analysis is correct. That is because all of the likely errors in the analysis would produce large artificial stress components. For example, if the three analyzed reflections come from different cell interiors, the corresponding angular misorientations of the cells would be misinterpreted as elastic distortions of the unit-cell geometry, resulting in large artificial elastic strains and stresses. This condition was artificially produced and the results are shown in Fig. 6 ▸. Here, three variants of the data from approximately 17.5 µm deep (see Table 1 ▸ and Fig. 5 ▸) are displayed. Variant A is the as-measured result, variant B was obtained by shifting the peak on the orange detector in the *X* direction by an amount comparable to the position difference of nearby peaks B and C in Fig. 3 ▸, and variant C was obtained by shifting the same peak by the same amount in the orthogonal direction. As expected, the stress components for variants B and C show considerable distortion when compared with the as-measured variant A.

## 6. Summary and conclusions   

We described and demonstrated a new measurement capability of the microbeam diffraction instrument on 34ID-E at the APS, which allows the mean geometry and crystallographic orientation of a strained unit cell to be determined for submicrometre sample volumes within a complex three-dimensional microstructure. Given the unstrained lattice parameter, the full elastic strain tensor can be obtained. With additional information on the elastic constants, the full stress tensor can be found. The one-sigma uncertainties for all extracted parameters are determined using a Monte Carlo uncertainty algorithm. Presently, the analysis software is limited to cubic systems, but extending this to arbitrary lattice systems should be straightforward.

Our approach was demonstrated by extracting full stress tensors from individual dislocation cells spaced along the 50 µm length of a buried polycrystalline Cu-TSV. In all cases, the diagonal components were nearly equal in magnitude and much larger than the off-diagonal components, consistent with a largely hydrostatic stress state. We emphasize that it is not possible to draw comprehensive conclusions about the role of stresses in Cu-TSVs on the basis of seven measured stress tensors in a single Cu-TSV, and note that very small grains in the TSV may not be represented in this small sample.

The ability to measure full strain and stress tensors from submicrometre sample volumes within complex microstructures has broad applications within materials science. Stresses are a principle cause of device failure through processes such as delamination, inter- and intragranular cracking, fatigue, whisker growth, hillock formation and driven diffusion processes. With submicrometre X-ray diffraction, these critical processes can now be studied nondestructively within real-world devices such as microelectronic devices and microelectromechanical systems.

## Figures and Tables

**Table 1:** Ideal and measured subtended angles between the reflections labeled A on the three detectors in Fig. 3 ▸, along with the residuals All uncertainties are one standard deviation.

Reflections	Ideal angle ()	Measured angle ()	Difference ()
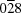 to 046	47.726	47.750.01	0.020.01
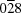 to 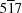	35.692	35.700.01	0.010.01
046 to 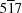	52.520	52.530.01	0.010.01

**Table 2:** Unit cell, orientation, strain tensor and stress tensor components from a sub-micrometre sample volume approximately 17.5m deep within a Cu-TSV The one-standard-deviation uncertainties were calculated using a Monte Carlo uncertainty algorithm as described in the text.

Unit-cell parameters (nm, )	
*a*_1_	0.3614970.000013
*b*_1_	0.3615580.000037
*c*_1_	0.3616560.000017
	90.00280.0097
	89.99880.0043
	89.99270.0049
Orientation ()	
	324.43010.0093
	149.20130.0048
	84.3410.013
Infinitesimal strain tensor components	
*e*_11_	(0.040.36) 10^4^
*e*_22_	(1.71.0) 10^4^
*e*_33_	(4.420.47) 10^4^
*e*_23_	(0.240.85) 10^4^
*e*_13_	(0.110.38) 10^4^
*e*_12_	(0.630.43) 10^4^
Stress tensor components (MPa)	
*s*_11_	7620
*s*_22_	8324
*s*_33_	9622
*s*_23_	413
*s*_13_	1.65.7
*s*_12_	9.66.5
